# Machine Learning Models with a Reject Option to Minimize Prediction Error: Application to Optical Properties of Dye Molecules

**DOI:** 10.21203/rs.3.rs-9649000/v1

**Published:** 2026-05-19

**Authors:** James Wellnitz, Travis Maxfield, Matthew Hart, Marielle Rath, Kathryn Kirchoff, Konstantin Popov, Alexander Tropsha

**Affiliations:** University of North Carolina at Chapel Hill; University of North Carolina at Chapel Hill; University of North Carolina at Chapel Hill; University of North Carolina at Chapel Hill; University of North Carolina at Chapel Hill; University of North Carolina at Chapel Hill; University of North Carolina at Chapel Hill

## Abstract

Predictions of the optical properties of dyes must be exceptionally accurate because even a small prediction error of, for instance, the wavelength can dramatically misrepresent the dye visible color. To address this challenge, we developed a novel model dubbed *‘CasRidge’*, which implements a machine learning concept known as reject option modeling. This model both predicts the target property(s) and provides the prediction confidence score. We show that by setting a threshold to reject predictions with confidence below a certain level, we improve the overall model accuracy. We benchmarked our model against several existing optical property prediction models, both with and without the reject option policy. We observed a significant performance improvement for most optical properties of dyes when using the reject option policy. As part of methodology development, we have explored the correlation between our learned confidence score and the traditional cheminformatics concept of “applicability domain”. We posit that the CasRidge approach can be employed for predicting any target property where very high prediction accuracy is especially critical. We dedicate this study to Dr. Terry Stouch, who always promoted high quality research.

## Introduction

High prediction accuracy is a natural objective of any computational modeling study attempting to predict chemical or materials property from their structure. Rapid growth of materials properties databases prompted extensive efforts to develop Quantitative Structure-Property Relationship (QSPR) models using diverse machine learning (ML) techniques and enhance predictive accuracy of these models using statistical validation approaches[1]. However, different areas of research may tolerate different levels of accuracy for the property prediction models. For instance, in biological high-throughput screening, where thousands of molecules can be tested for the desired activity rapidly and inexpensively, lower model accuracy can be acceptable[2]. On the contrary, there are areas of research where there is an expressed need to achieve supreme accuracy in predicting target materials properties because of the high cost or low speed of testing, and/or due to the type of the property of interest. One such area is predicting optical properties of solvent-dye mixtures, where the development of models with exceptionally high predictive accuracies is crucial because even small error will result in inaccurate prediction of the dye color. This study explicitly addresses the latter challenge by developing a novel methodology that we dubbed “CasRidge”.

Dyes and colorants are a ubiquitous part of human life. Natural dyes have been in use for the better part of 5,000 years [3], [4], [5]. The advent of modern synthetic chemistry led to the development of artificial dyes, enabling a host of vital technologies such as bioimaging[6], solar cell construction[7], photocatalysis[8], as well as bio-based[9], physics-based[10], materials-based[11], and food-based[12] applications[13]. There is a deep interest in developing dyes not only as colorants, but as technological components, which has driven the need for dyes that exhibit specific properties, tailored for certain applications. Given the diversity of organic dye chemical space[14], trial-and-error experimental discovery and human intuition are insufficient for the exploration of every possible combination of organic dye and dye-based technology[15]. Computational methods are required to assist in the discovery process. To be effective, such methods need to be designed to account for the role solvents play in a dye’s optical properties, known as the solvatochromic effect[16]. Methods also need to achieve exceptionally low error in prediction, because, as briefly alluded to above, even a slight error can result in dramatic difference for a dye’s colors or optical properties between prediction and experiment.

In the recent past, *ab-initio* methods have been commonly utilized in the field of rational dye design[7], [17]. Methods including time-dependent density functional theory (TDDFT)[18] have allowed for the prediction of the absorption and emission spectra of organic molecules[19], the main properties determining the color of a dye[20]. However, despite the accuracy achieved by quantum mechanical methods, there are still drawbacks of these approaches. TDDFT has been known to be particularly time-consuming and computationally expensive, with some calculations for a single compound requiring up to three days to complete[21], limiting their usability in large scale screening of candidate molecules.

Statistical and ML modeling have offered a viable alternative to *ab-initio* methods[22]. With sufficient amounts of data, ML based methods have been shown to approach DFT-like accuracy[23]. Previous studies by Joung et al[24], Ju et al[25], and Greenman et al[26] demonstrated the capability of different ML algorithms to predict various optical properties of dyes with appreciable accuracy. Ju et al[27] employed chemical fingerprints in tandem with a gradient boosted tree (GBT) model. Both Joung et al[24] and Greenman *et al* utilized neural networks: Joung et al. implemented an undirected message passing network while Greenman et al. implemented a directed message passing network[28] to develop a method they called ChemProp.

While these previous models achieved good accuracy, there is still a need for improvement. The primary reason, as mentioned earlier, is that dye properties, and optical properties in particular, need to be predicted quantitatively and with exceptionally high accuracy. For example, even a shift in the maximum absorption/emission wavelength (abs-λ_max_) by just a few nanometers could have a significant impact on the dye’s color. This can lead to the costly synthesis and characterization of molecules unsuitable for specific applications—such as serving as a green light emitter in OLED applications—due to unwarranted confidence in the model’s results. To attain such a heightened level of prediction accuracy, it is critical to employ special methodologies. For example, concepts like mixture-aware model validation[29] and the “applicability domain”[30] can be employed to assess whether the chemical structure of a given molecule deviates too significantly from the structures in the training set, possibly leading to unreliable predictions.

**Figure 1:**
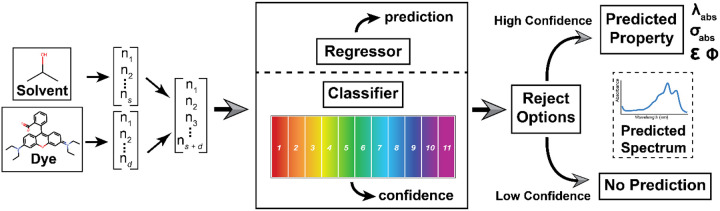
Overview of the machine learning with a reject option approach used to predict dye optical properties. First, dyes and solvent molecules are featurized separately and then respective features are combined. The joint features are passed into a regression model for the optical property prediction and a classification model for the confidence value prediction. The confidence value is used to either accept or reject the prediction. If all required properties are predicted, the absorption (or emission) spectrum can be estimated with high accuracy and confidence.

In this study, we present a novel modeling approach with a primary focus on achieving an extremely high prediction accuracy for dye optical properties. Specifically, we have devised a unique model architecture termed “CasRidge”. This approach integrates a reject option policy[31] into the ML model, resulting in the generation of a highly reliable confidence level associated with each prediction (Fig. 1). The reject option methodology allows for the potential rejection of predictions for individual molecules if the associated confidence level assigned by the model is low. Ideally, an impeccable reject option model would exhibit a perfect correlation between the highest confidence levels and the most accurate predictions, while associating the lowest confidences with the least accurate predictions, arranged in a ranked-order manner. By adjusting the confidence threshold, one can limit the number of predictions made (often referred to as model coverage), while simultaneously enhancing the accuracy for molecules that surpass the confidence threshold. This dual enhancement in both precision and selectivity underscores the potential of our approach to transform the landscape of dye optical property prediction and significantly augment its real-world applicability.

We have conducted an in-depth exploration of the CasRidge model performance both with and without the reject option policy turned on. To facilitate a comprehensive comparison, we have integrated the reject option model into previously established methodologies. Furthermore, as a rigorous method to assess model performance, we employed a recently proposed mixture validation approach[29] that, to the best of our knowledge, has not been utilized in other studies. We employed a mixture modeling and validation framework mindful of the fact that optical properties of dye molecules have been typically assessed in the presence of solvent. It has been shown that solvents can affect property measurement and thus should be accounted for in our study design by calculating chemical descriptors of both dyes and solvents. By adopting this approach, we could evaluate the performance of our new models under conditions that closely resembled their real-life applications. Moreover, this method effectively eliminates unintended data leakage between training and validation data,[29] guaranteeing the unbiased accuracy of model performance statistics.

Our findings suggest that the integration of the reject option policy with CasRidge leads to higher accuracy predictions for the optical property endpoints compared to other models, particularly when utilizing a high confidence threshold. Furthermore, in the broader sense of ML modeling of any experimental data, we expect that the CasRidge architecture could have wide applications in fields where prioritizing prediction accuracy is crucial, even at the cost of the reduced model coverage. Such a strategy would especially mitigate the risk of synthesizing compounds with undesirable properties.

We dedicate this paper focusing on the critical issue of QSAR/QSPR model prediction accuracy to Dr. Terry Stouch, one of the true leaders in computational chemistry and statistical QSAR molecules. The senior author of this contribution, Prof. Alexander Tropsha, has benefited for many years from Terry’s quiet wisdom, advice, and support. Terry’s paper on “In silico ADME/Tox: why models fail” in this journal in 2003,[32] his second most cited paper, was especially influential in my critical thinking about challenges and needs for best practices in model development that we ended up developing.[33] We consider studies reported in this paper an important addition to current practices of QSAR/QSPR model development, especially, when very high accuracy is critical as is the case in dye color prediction. We hope that this contribution will be a genuine tribute to Terry’s unique sense of quality research.

## Results

### Baseline model performance

The initial step in our evaluation process involved assessing model performance without the inclusion of the rejection model. This allowed us to establish a baseline performance for three distinct model architectures to predict six optical property endpoints: wavelength of maximum absorption (*abs-λ*_*max*_), wavelength of maximum emission (*emi-λ*_*max*_), log photoluminescence quantum yield (*log PLQY*), absorbance bandwidth (*abs-σ*), emission bandwidth *(emi-σ*), and log extinction coefficient (*log ε*). Two of these models, Gradient Boosting Trees (GBT) and ChemProp[34], had been studied previously in the context of predicting the maximum wavelength of absorption[25], [26], whereas the third, CasRidge, is a cascading-based model introduced in this study (See Methods). To compare our CasRidge to previous methods, we re-implemented and re-evaluated the previous modeling approaches for all six endpoints using a mixture validation approach.[29]

In a baseline performance test involving four out of the six endpoints, all three ML models demonstrated nearly equal performance on the chromophore-out validation splits (Table 1). At the same time, the ChemProp model slightly outperformed GBT and CasRidge for emi-λ_max_ and abs-λ_max_. In addition, evaluation of model performances on the absolute-out validation splits found that across all three models, the overall performance on the endpoints decreased when we employed the absolute-out validation splits instead of the chromophore-out validation splits. It is important to note that the models and training sets remained identical between both validation approaches. Therefore, any difference in performance can be attributed solely to the variance in the validation sets. Specifically, two endpoints, abs-λ_max_ and emi-λ_max_, exhibit the most significant differences between the absolute-out and chromophore-out validation approaches, suggesting potential data leakage from solvent duplicates in the test and training sets. Our analysis indicates that the accuracy of the models for specific endpoints can vary by as much as 12%, depending on whether a particular solvent was part of the training set. This again confirms the critical importance of context-aware validation techniques when working with mixture data to prevent the mischaracterization of model performance in specific tasks.

We compared ChemProp, GBT, CasRidge and the Deep4Chem model[24], which had been previously reported for predicting the same six endpoints. However, it is important to note that the Deep4Chem model lacked validation in a mixture-aware fashion, and the code for its reimplementation is unavailable, which somewhat limited the value of our comparisons. To overcome this issue, we carried out random (mixture-unaware) validation, similar to the approach used for Deep4Chem, with all three models. The results indicated that all three models outperformed Deep4Chem for four of the endpoints, while the remaining two endpoints, namely log PLQY and log ε, exhibited comparable performance (see **Table S2)**. Importantly, these findings highlight that the full coverage baseline models already demonstrate competitive performance within the context of random validation, positioning them favorably for state-of-the-art performance.

## Modeling with reject option

**Figure 2:**
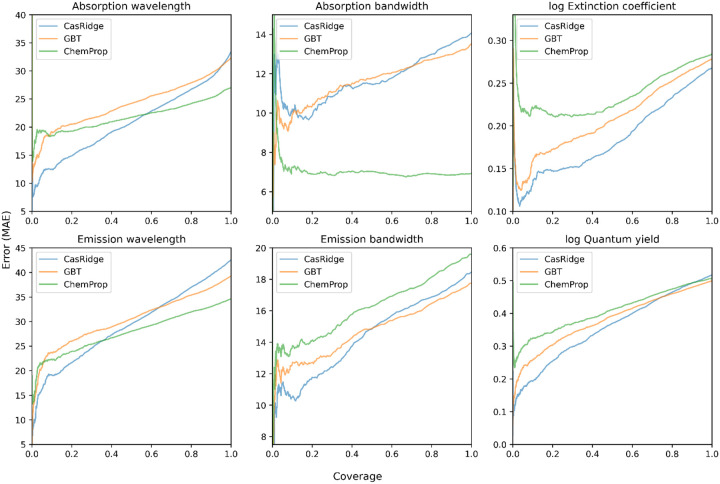
Coverage-error curves for each endpoint for CasRidge (blue), GBT (orange) and ChemProp (green). Mean absolute error (MAE) is in respective endpoint units using chromophore-out cross validation. Coverage is the percentage of endpoint predictions not rejected, e.g., a coverage of 1 means no data points were rejected. As coverage decreases so does the number of data points used to assess the MAE, which can cause artifacts at low levels of coverage, as observed in some endpoints.

Each of the three model architectures (ChemProp, GBT and CasRidge) were evaluated with chromophore-out splits utilizing the additional reject option model to limit predictions with low model confidence. Reject option modeling takes as input a confidence threshold below which predictions are ignored (or rejected). The proportion of data points whose predictions are *not* rejected is called the coverage of the model. Model performance, characterized by the mean absolute error (MAE) of the predictions, as a function of the coverage (termed coverage-error curves) can be used to assess general performance of the reject option model and is illustrated in Fig. 2. At higher coverage levels, meaning fewer data points are rejected, both ChemProp and GBT exhibit better performance for most endpoints, with the exception of log ε. However, at lower coverages, the CasRidge model outperforms the other two models for all endpoints except abs-σ (nm). CasRidge performed exceptionally well at 25% coverage, with MAE reduced by approximately two-fold compared to baseline for four of the endpoints (Table 2). All the models showed significantly superior performance compared to the random rejection option controls. **(Figure S1)**.

**Figure 3:**
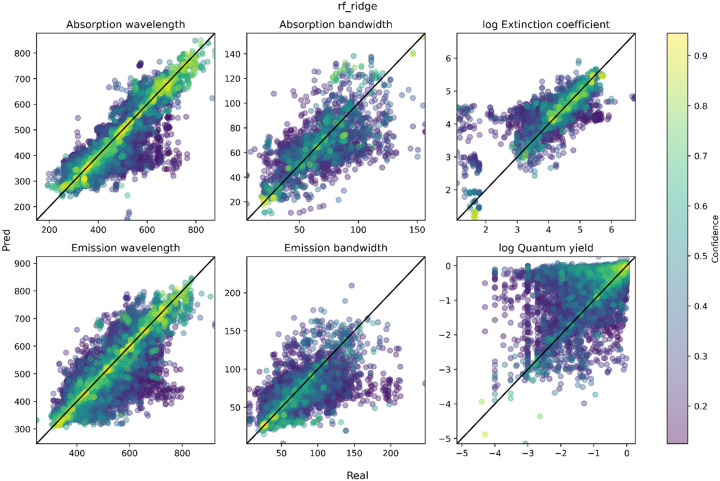
Scatterplot of predicted vs. experimental values for each of the six endpoints using the chromophore-out validation set. Each prediction is colored according to its confidence, the output of the reject option model, a score ranging from 0 to 1. We see that high confidence values tend to fall near the diagonal line representing predictions with no error.

In Fig. 3, the relationship between the reject option model confidence and prediction accuracy is depicted for all endpoints. Notably, there exists an inverse relationship between confidence and error in predictions generated by the CasRidge reject option model. This means that predictions assigned high confidence by the reject option model consistently exhibit low errors. However, the reverse is not universally true as the rejection model did not display a similarly strong connection between low confidence and high error, as illustrated in **Figure S2**. Consequently, a prediction with high confidence is likely to have low error, but a prediction with low confidence may yield either a small or a large error.

Both ChemProp and GBT demonstrated similar results to those of CasRidge, albeit their capacity to establish a strong relation between high confidence and low error appears weaker compared to CasRidge (**Figure S3**). Similar to the baseline evaluation, the reject option model using absolute-out splits exhibited slightly worse overall performance in comparison to the chromophore-out splits (**Table S3**). Notably, the validation using absolute-out splits for the two bandwidth endpoints displayed poorer performance, likely attributable to the limited amount of available data for these specific endpoints.

## Comparison with quantum mechanical calculations

Computational quantum mechanics (QM) methods can be employed to predict two out of the six endpoints of interest: the wavelength of maximum absorption and the wavelength of maximum emission. We compared performance between TDDFT, semi-empirical and ML methods for estimating these two endpoints. Both QM methods exhibited inferior performance even when compared to the best 100% (full) coverage ML model, selected from CasRidge, GBT, and ChemProp. Furthermore, the performance of QM-based calculations was substantially worse when compared to the low coverage ML models (Table 3).

Both QM methods also exhibited significantly slower computational times compared to ML approaches. Calculations utilizing semi-empirical methods required an average of 30 minutes per compound, while TDDFT could take up to 3 days per compound when running on a standard desktop computer. In contrast, all ML methods displayed an average computing time of under 60 seconds for approximately 5,000 compounds using the same computer. This stark contrast in computational efficiency further highlights the effectiveness of ML methods for large-scale predictions, offering both high accuracy and significant time savings.

## Color case study

For both absorption and emission, Curcumin and Rhodamine B had relatively high confidence values averaged across all relevant endpoints compared to the much lower confidence for Reichert’s Dye. Curcumin and Rhodamine B also had accurate color predictions for both emission and daylight, a result that matches expectations given their higher confidence. Reichert’s Dye was far off on its color predictions, again matching the expected outcome given the low confidence assigned. Using this color prediction approach alongside the models allows for dye/solvent pairs with desired colors to be quickly and accurately screened, which can provide a major boost for novel dye discovery campaigns.

**Figure 4:**
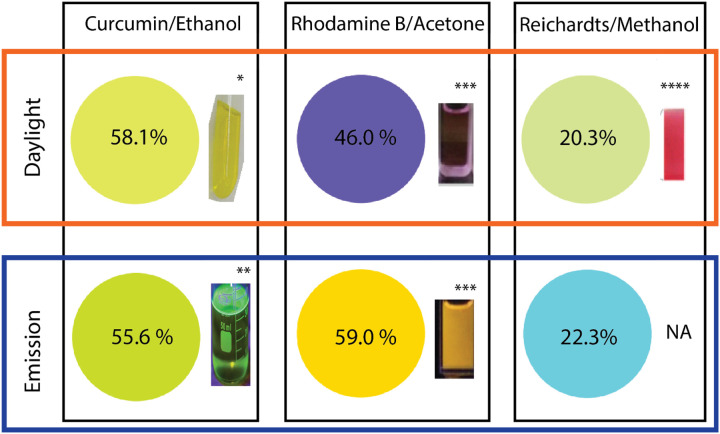
Predicted daylight and emission colors for curcumin in ethanol, rhodamine B in acetone and Reichert’s dye in methanol. Predicted colors are displayed as solid circles with the overall confidence of the color prediction present in the center. Reference images of true color are to the right of each prediction, with the exception on Reichert’s emission, where no reference image was found in literature. *From[35]; **from[36]; ***from[37]; ****from.[38]

By utilizing these six predicted endpoints, we can estimate the color of emission and the color in daylight for dye/solvent pairs. We applied this method to three specific dye/solvent pairs: Curcumin in ethanol, Rhodamine B in acetone, and Reichert’s Dye in methanol (Fig. 4). It is important to note that these dyes were either withheld from or not present in the training set used to construct the model. The predictions for both Curcumin and Rhodamine B displayed relatively high confidence values in contrast to the significantly lower confidence observed for Reichert’s Dye. As a result, calculations for Curcumin and Rhodamine B yielded precise color predictions for both emission and daylight, whereas predicted colors for Reichert’s Dye were significantly off (Fig. 4). Leveraging this dye color prediction approach in combination with our proposed modelling techniques facilitates rapid and accurate screening of dye/solvent pairs for identification of desired colors. This capability holds the potential to provide significant advantages for novel dye discovery initiatives.

## Discussion

### Context of use for reject-option modeling with CasRidge

The results have shown that utilizing reject option with CasRidge can produce a final set of accepted predictions with improved accuracy compared to no rejection. This comes at the cost of limited coverage: losing the ability to get a prediction for all compounds. Further, the most significant gains in performance are generally observed at low (< 20%) coverage. This is likely due to generally limited ability of machine learning models to extrapolate far outside the training data, a phenomenon commonly known as applicability domain. CasRidge is likely to be further hindered in this use case by also having limited and potentially noisy data available for training, resulting is a vast majority of predictions being of low confidence. This creates a potential issue when using the CasRidge for small scale prediction on prioritizes chemical candidates. For example, if 100 compounds have already been prioritized and predictions are required for all 100, CasRidge, or any reject-option modeling is unlikely to be useful. However, if the task is to discover a few chemicals of interest with desired properties from a large set of potential compounds, CasRidge is likely to give better results, as in this case it is okay to reject most predictions given that the goal is not to find *all* compounds.

This analysis suggests a new hyperparameter to be tuned when using CasRidge: the desired coverage/error ratio. In all cases of use, the ideal value for this trade off ratio will be different. To best determine it, several cross-validation runs, like those done in this study should be conducted with the training data to produce coverage/error curves. Using this curve and the context of use, an acceptable coverage/error ratio can be selected and the confidence cutoff to generate that ratio selected using the curve. In our case, we observed acceptable coverage/error at confidence levels above 75%.

## Learned confidence compared to chemical similarity

**Figure 5:**
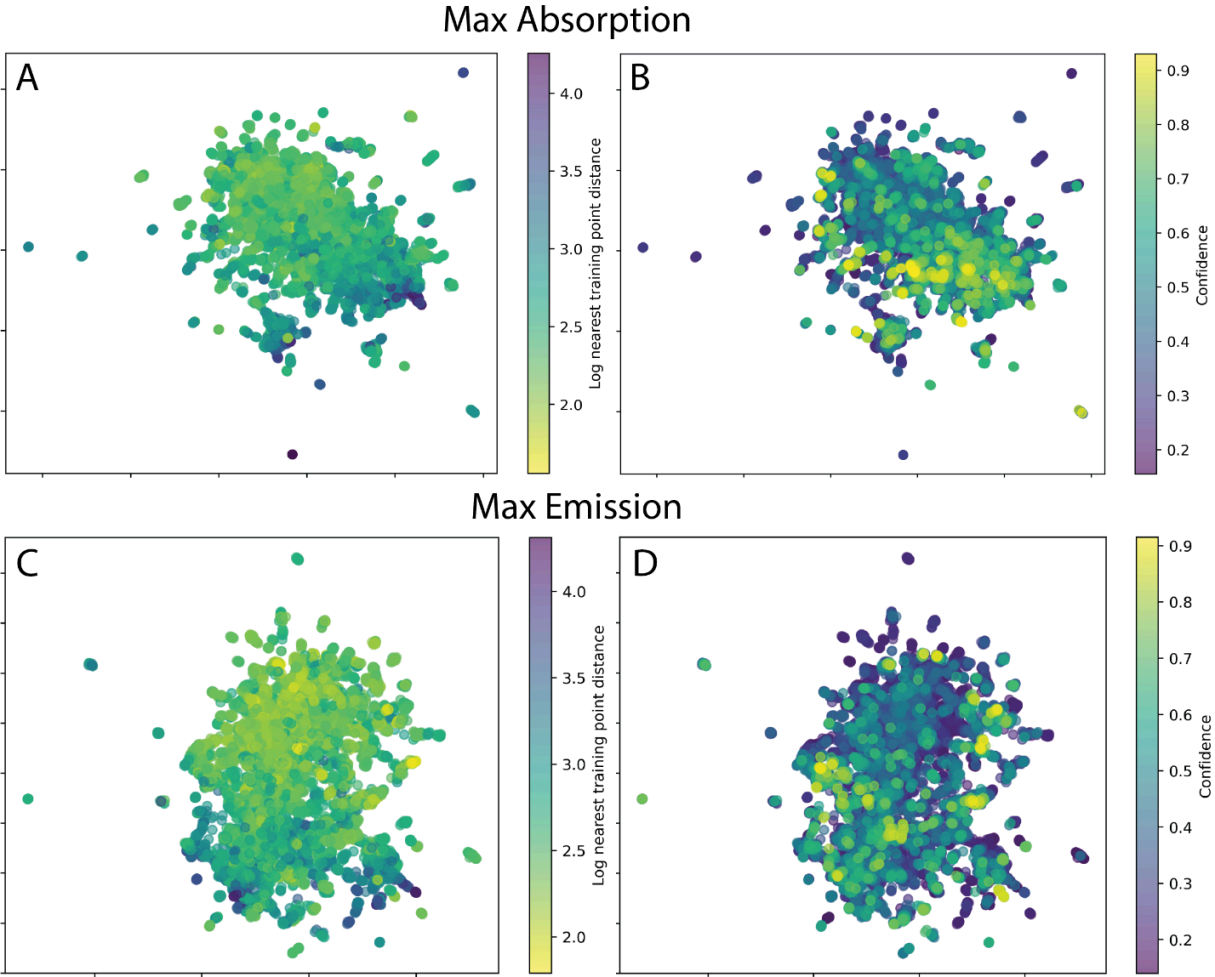
Two-dimensional rendering of chemical space of abs-λ_max_ (top) and emi-λ_max_ (bottom) endpoints colored by: A&C – similarity between the datapoint and the training set, where similarity is defined as the smallest Euclidean distance between the chemical descriptors of the datapoint and all training data; B&D – confidence assigned to that point by the rejection model. The color scale for A&C was inverted such that colors among all plots both hold the same meaning (yellow is high, purple is low). Two-dimensional representations were generated using Umap.[40]

One may hypothesize that the reject option model is simply assigning high confidence to dye-solvent pairs most similar to those previously observed during training. Therefore, using simple and quick chemical similarity-based metrics to reject predictions, commonly used in cheminformatics for assessing model applicability domains[1], [30], [39], could be as effective for improving accuracy on high confidence predictions compared to our learned model. If this hypothesis were true, the distribution of chemical similarity and rejection confidence should be highly correlated across the chemical space. That is, areas where rejection confidence were high should also show *low* chemical similarity to the training set. We found that the two distributions are in fact different (Fig. 5, **Figure S4)**. The similarity distribution exhibits a more global pattern with a gradual gradient across chemical space, while the rejection confidence displays discrete pockets of confidence dispersed throughout the entire space. These findings suggest that the models’ performance at assigning prediction confidence is not merely defined by chemical similarity between training and test sets. Instead, the reject option model appears to have learned a policy that was able to take advantage of other “hidden” trends in the data not available to conventional chemical similarity-based rejection. We also found that unique set of predictions the chemical similarity approach suggests will have high error (e.g. less chemically similar, higher error) poorly correlated with prediction error and inferior in correlating prediction confidence compared to CasRidge **(Figure S2, Figure S5).** Together these results suggest the potential for moving beyond a simple chemical similarity calculation to assess a model confidence in a prediction.

## Choosing appropriate validation techniques

The selection of validation techniques holds critical importance in the development and performance assessment of any ML model. Inadequate validation protocols can result in overly optimistic performance metrics that do not accurately reflect a model’s real-world performance when dealing with new, untested dye molecules. Such a scenario can render the modeling predictions essentially useless, thereby resulting in unexpected experimental outcomes. For example, the dataset used in this study requires extra caution during validation, as each datapoint represents a binary mixture that consists of two components: a dye and a solvent. This situation introduces the possibility that a specific mixture component, such as the same dye with different solvents, could exist in both the training and validation datasets, representing a potential instance of data leakage. Failing to adequately account for this possibility can lead to the generation of artificially inflated performance metrics[29]. Consequently, meticulous attention to validation techniques is especially imperative to ensure the model’s true predictive capacity in scenarios involving mixtures like those in this dataset.

In the dye-solvent dataset used in this work, a significant source of potential data leakage comes from the dye components. In fact, for each endpoint, approximately 33% of the dyes are duplicated across different solvents (**Table S4**). While the solvatochromic effects can reduce the data leakage due to slight shifts in optical properties for the same dyes in different solvents[16], these shifts, for the solvents in the dataset, tend to be relatively minor and therefore do not affect the data leakage problem **(Figure S6)**.

In scenarios where data leakage is left unaddressed, such as in the random split validation approach employed by previous models discussed in this work, there is a significant increase in validation performance (**Figure S7**). However, this inflated performance does not accurately reflect the true accuracy of the model in its intended purpose: predicting properties of dyes that the model has not been trained on. This aligns with findings and comments by Greenman *et al*[26]. While less significant than duplicate dyes, we also observed the potential for data leakage from duplicate solvents in both training and test sets. Notably, the chromophore-out validation method, focusing solely on preventing duplicate dyes between training and test sets, outperformed the absolute-out approach, which also prevents duplicate solvents (Table 1). Importantly, prior studies on dye modeling did not account for the possibility of solvent-related data leakage. Neglecting this potential leakage, as likely to be the case in random and even the chromophore-out split validations, is inappropriate when assessing model performance. To ensure more accurate evaluations, it is imperative to employ rigorous mixture validation approaches.

At the same time, we recognize that the leakage of a particular mixture component does not always pose a problem. Such leakage becomes an issue only when the model is expected to make predictions on data points containing component compounds that were not part of the training set. Therefore, whether leakage due to solvents, dyes, or both needs to be addressed during validation hinges on the intended use of the model. For example, when predicting optical properties for dye-solvent mixtures, the models are likely to be used with new dyes. Thus, dye data leakage during validation is a concern. In contrast, using new solvents is less common so accounting for solvent data leakage is not always necessary. Therefore, we suggest two validation approaches that mirror real use cases: one addressing only dye leakage (no new solvents) and one accounting for both dye and solvent leakage (new solvents allowed), corresponding to the chromophore-out and absolute-out validation splits, respectively (See Methods).

## Conclusion

The primary limitation of the reject option modeling approach arises when a high confidence threshold is selected, resulting in a considerable number of data points lacking associated predictions. This situation can potentially lead to the rejection of a compound possessing the desired optical properties, merely because the model was not able to assign it with a sufficiently high level of confidence. Furthermore, as observed in this study, reject option policies are not infallible and may occasionally reject compounds with even low prediction errors.

Interestingly, these apparent limitations can also be viewed as advantages. In fact, this approach has the significant benefit of markedly reducing the false positive rate in predictions. Selections based on these predictions are more likely to align closely with experimental measurements. Consequently, in scenarios where high false positive rates come with substantial costs, such as in discovery campaigns that involve the synthesis and testing of novel chemical matter, the reject option emerges as a powerful tool for cost reduction. Notably, these conclusions are applicable to a large range of problems beyond dye modeling, which is why we posit that methods developed and employed in this work should find broad application in many areas of chemical and materials discovery research.

## Methods

### 1. Dataset collection and curation

An original dataset containing 20,236 dye-solvent combinations with known molecular structures and their respective experimentally measured endpoints was taken from Joung et al[41] Most data entries included both a dye and a solvent, where the original dataset included a solid matrix formally defined as solvent. Some dyes were present in the dataset without a solvent, i.e., in solid state. We removed all data entries without a solvent, leaving 19,261 data points. Furthermore, we removed all duplicate dye-solvent pairs, regardless of endpoint agreement, as we expected these to be the result of a data processing error. Lastly, we removed all data points containing invalid SMILES strings as determined by RDKit software[42]. This curation effort left a dataset of 18,606 valid data points, consisting of the combinations of 6,680 unique dyes and 382 unique solvents.

Many of the 382 solvents were only observed in combination with a single dye. These data points present limited information and increase the complexity of the dataset. To address this limitation, we removed all data points including a solvent that occurred less than 100 times in the entire dataset. This requirement reduced the number of solvents in our dataset from 382 to 22 (**Table S5**) but did not significantly reduce the size of the overall dataset. The remaining set of samples including one of the 22 common solvents comprised 90.6% of the original dataset, containing 16,866 data points and 6,539 unique dyes.

Of the seven optical properties presented in the original dataset, we considered only the six needed to estimate the absorption and emission spectrum: the wavelength of maximum absorption (abs-λ_max_), absorption bandwidth (abs-σ), extinction coefficient (ε), wavelength of maximum emission (emi-λ_max_), emission bandwidth (emi-σ), and photoluminescence quantum yield (PLQY). PLQY and ε span several orders of magnitude which could make model development more difficult; thus, their log transformations were used instead: Log PLQY and Log ε, respectively. Not all dye-solvent pairs had measurements for every endpoint; many included the wavelength of maximum absorption and/or emission, but not the other four endpoints. In fact, approximately 85% of possible measurements were missing from the dataset. In the end, we created a separate dataset for each endpoint by selecting only the data with experimental measurements for the given endpoint, resulting in six distinct datasets of varying size to be used for model development (**Table S6**).

### 2. Featurization of molecules

The inputs to our models were dye–solvent combinations. To featurize these bi-molecular entities, hashed (non-binary) extended-connectivity fingerprints (ECFPs),[43] implemented in RDKit, were computed for each dye-solvent pair as follows: an ECFP with radius 4 and 2048 bits was calculated for the dye, and an ECFP with radius 3 and 256 bits was calculated for the solvent. The two fingerprints were concatenated, resulting in a feature vector with 2,304 bits for a single dye-solvent combination. The fingerprints used herein accounted for chirality.

### 3. Discrete labeling

All endpoint measurements were originally continuous values. However, the CasRidge and reject option model implemented here (see below) require discrete class labels. To generate discrete classes, continuous data for each endpoint were discretized to a set of N ordinal classes, corresponding to N intervals of equal size, ranging between the 5th and 95th percentiles of the respective property value distributions. Two additional classes were then defined as *below the 5th* (underflow) or *above the 95th* (overflow). For all endpoints we used 9 fixed-size ranges (N = 9) and the overflow and underflow classes, resulting in 11 possible discrete classes.

### 4. Regression modeling

#### 4.1 CasRidge

**Figure 6:**
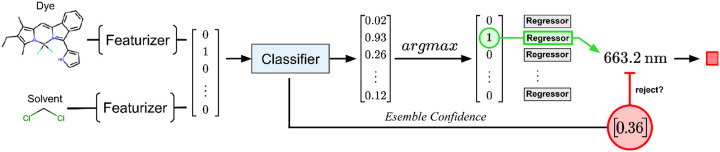
Description of the CasRidge model architecture used to predict the optical properties of dye solvent mixtures with a reject option.

The cascading ridge regression (CasRidge) model is built following the cascading architecture approach[44] (Fig. 6). Each CasRidge cascading model consisted of two layers: (1) a random forest classifier[45] and (2) several ridge regressors[46], one for each of the possible discrete classes from the classifier. Thus, our use of 11 discrete classes required each cascading model to be composed of (1) a single random forest classifier and (2) 11 ridge regressors. The random forest classifier was trained on featurized dye–solvent pairs, as detailed in Methods Sec 2, to predict discretized endpoint values, as detailed in Methods Sec 3. Separately, the several ridge regression models making up the second layer were trained using only data points in the assigned discrete class with continuous labels. When used for prediction, the model first predicts probabilities for each discrete class and then employs the class-specific Ridge model corresponding to the class with the highest probability to generate a continuous prediction.

The use of discrete boundaries can result in the model struggling with data points that fall near a given boundary. To address this issue, CasRidge used an ensemble of cascading models, with the number of models here denoted as **C**, each trained on the same data, but with different discrete class definitions and boundaries. The first of the **C** ensemble of models was defined according to the discretized endpoint boundaries described in Methods Sec 2. Then, for each of the subsequent models, the discrete class boundaries were shifted to the right relative to the previous model by a fixed value, equal to the fixed size of the classes divided by **C** – 1, resulting in **C** discrete class definitions whose class boundaries are not identical. When employed for prediction, the ensemble predicts **C** class probabilities and **C** continuous predictions for a given data point. The **C** class probabilities are subsequently passed through a SoftMax function to convert them into weights for each of the **C** continuous predictions. Mathematically, the SoftMax function exponentiates the input values, making them positive, and then normalizes them by dividing them by the sum of all exponentiated values. As a result, the output values fall between 0 and 1 and sum up to 1, forming a valid probability distribution. The SoftMax is often used in ML and statistics for multi-class classification problems, where it can convert raw scores or logits into probabilities (here referred to as weights) that indicate the likelihood of each class. These weights are then used to calculate the weighted average of **C** continuous predictions — multiplying each prediction by its associated weight and summing. This sum is returned as the final continuous prediction. The models developed herein used an ensemble size of 4, i.e. **C** = 4.

#### 4.2 Gradient boosting trees

The gradient boosting tree regressor (GBT) [47] was employed as implemented in Sci-Kit Learn [48] and used the hyperparameters outlined in Ju *et al*[25]. GBT models used the featurization described in Methods Sec 3 to input dye-solvent combinations.

#### 4.3 ChemProp

The ChemProp model[28] was implemented from https://github.com/ChemProp/ChemProp. This approach employs Direct Message Passing Neural Networks architecture. Hyperparameter tuning was done with initial values taken from Greenman *et al*.[26] ChemProp does not require a manual featurization step, thus the input to the ChemProp models is the SMILES[49] of the dye and solvent.

### 5. Reject option modeling

The reject option model was built as an ensemble of **C** random forests, implemented and trained the same way as layer (1) from Methods Sec 4.2. — using discrete classes in the ensemble approach. During predictions **C** confidences are produced. These confidences are converted into weights using a SoftMax function. These weights are used to calculate the weighted average of the **C** confidence values, generating a single confidence associated with a given datapoint. The reject option model herein uses an ensemble size of **C** = 4.

For the ChemProp and GBT models, the reject model was trained separately in parallel, yielding two distinct models: one for continuous prediction and another for rejection. In contrast, for the CasRidge the reject option model is built-in as the first layer (1) of each cascading model. Consequently, a single CasRidge model can act as both the continuous model and the reject option model depending on how the outputs of the model are processed.

Rejecting certain predictions inevitably results in decreased coverage, corresponding to the proportion of data with accepted predictions. To serve as a control comparison for our reject option models, we employed a random, coverage-matched reject policy for each of the base architectures. In this approach, rather than rejecting predictions based on a confidence threshold, we randomly discarded predictions until achieving a matching coverage level.

### 6. Validation split generation

Due to the solvatochromic effect, our dataset of dye-solvent pairs is best modeled as a mixture dataset. Best practices in QSAR modeling of mixture datasets[29], require stratifying a mixture model’s validation into one or several mixture-aware validation splits. Validation splits for mixture models are designed to prevent the same mixture components from appearing in both the training and validation datasets. In our context, these components encompass both the dyes and solvents. Notably, we deviate slightly from the procedure described in Maxfield *et al*[29] by not considering individual dye molecules for separation, but rather their Murcko scaffolds[50], which we refer to as chromophores. This adjustment further strengthens the separation between the training and validation datasets. With this in mind, we construct four splits: the training split, a random collection of dye-solvent compounds; the chromophore-out split, a validation set consisting of dye-solvent compounds whose dye components do not share scaffolds with those dyes present in the training set; the solvent-out split, consisting of dye-solvent compounds whose solvent components are not present in the training set; and the absolute-out split, consisting of dye-solvent compounds where neither the dye scaffolds nor the solvents are present in the training set. Our models are validated using the chromophore-out and absolute-out splits.

### 7. Color Prediction

We adopted the color prediction approach outline in Joung et al[24] Specifically, we modeled absorption and emission spectra as single, Gaussian peaks (see Eq. 1 from Joung et al[24]) and converted to RGB values using the 1931 CIE color space. To model the color of a sample illuminated by daylight, we used the CIE Standard Illuminant D65 and assumed a standard 1cm deep vial.

## Supplementary Material

This is a list of supplementary files associated with this preprint. Click to download.

- supplementalJCAMD.docx

## Figures and Tables

**Table 1:** Mean absolute error (MAE) of the regression models using absolute-out (Abs) or chromophore-out (Chrom) cross-validation for all six endpoints (columns). Additional data can be found in Table S1.

Model - Validation	abs-λ_max_ (nm)	emi-λ_max_ (nm)	abs-σ (nm)	emi-σ (nm)	log PLQY	log ε (M^−1^·cm^−1^)
CasRidge - Abs	38.9 ± 5.4	51.45 ± 5.04	15.06 ± 1.24	21.9 ± 2.7	0.61 ± 0.06	0.29 ± 0.04
CasRidge - Chrom	33.44 ± 1.4	42.6 ± 0.62	14.15 ± 0.74	18.66 ± 1.64	0.52 ± 0.02	0.27 ± 0.03
GBT - Chrom	32.31 ± 0.67	39.21 ± 1.99	13.64 ± 0.98	17.95 ± 1.39	0.5 ± 0.01	0.28 ± 0.02
ChemProp - Chrom	27.02 ± 2.03	34.57 ± 2.37	6.97 ± 0.83	19.95 ± 2.29	0.51 ± 0.03	0.29 ± 0.03

**Table 2:** Mean absolute error (MAE) for the CasRidge reject option model at several coverage levels (row) for each of the six endpoints (column). Performance calculated using chromophore-out splits. The lowest MAE shown in bold.

Coverage	abs-λ_max_ (nm)	emi-λ_max_ (nm)	abs-σ (nm)	emi-σ (nm)	log PLQY	log ε (M^−1^·cm^−1^)
75%	25.69 ± 1.6	35.4 ± 1.04	12.69 ± 0.88	16.77 ± 1.6	0.45 ± 0.02	0.22 ± 0.03
50%	20.77 ± 1.2	29.39 ± 1.45	11.46 ± 0.71	15.08 ± 1.75	0.37 ± 0.03	0.18 ± 0.02
25%	**15.96 ± 1.88**	**22.94 ± 2.05**	**10.57 ± 1.25**	**12.33 ± 1.35**	**0.28 ± 0.05**	**0.15 ± 0.01**

**Table 3:** Mean absolute error (MAE) of TD-DFT, semi-empirical methods and best performing machine learning method (ML) in predicting the six endpoints. ML are from metrics from chromophore-out split validation.

TD-DFT	abs-λ_max_ (nm)	emi-λ_max_ (nm)	abs-σ (nm)	emi-σ (nm)	log PLQY	log ε (M^−1^·cm^−1^)
	53.93*	77.78**	NA	NA	NA	NA
Semi-empirical	146.21	162.45	**NA**	**NA**	**NA**	**NA**
Best ML Full coverage	27.02 ± 2.03	34.57 ± 2.37	6.97 ± 0.83	17.95 ± 1.39	0.5 ± 0.01	0.27 ± 0.03
Best ML 10% coverage	12.44 ± 1.55	19.0 ± 2.09	6.82 ± 1.89	10.37 ± 1.39	0.2 ± 0.01	0.13 ± 0.03

* *From Greenman *et al*.[26]

** **From Joung *et al*.[24]

## Data Availability

All data used for training is available at https://github.com/molecularmodelinglab/DyeRejectOption/tree/main/data. Scripts, source codes and full documentation for CasRidge can be found at https://github.com/molecularmodelinglab/DyeRejectOption.
